# Anxiety and depression during COVID-19 in Kosovo: literature review and analysis of repeated cross-sectional surveys

**DOI:** 10.1192/j.eurpsy.2025.1268

**Published:** 2025-08-26

**Authors:** N. Fanaj, S. Mustafa, E. Krasniqi

**Affiliations:** 1 Alma Mater Europae Campus College Rezonanca, Prishtina; 2 PMSH, Prizren; 3 AAB College; 4 UBT College, Prishtina, Kosovo

## Abstract

**Introduction:**

The literature review on anxiety/depression rates during Covid-19 is inconsistent due to significant methodological challenges in quantifying the impact of the pandemic on the mental health of the population.

**Objectives:**

To identify and understand the prevalence of anxiety/depression as a result of the COVID-19 situation aiming to synthesize impact of COVID-19 in mental health of population in Kosovo.

**Methods:**

It’s a classic literature review which involves a thorough examination of existing research in internet, including raw data of the individual empirical results from several repeated cross-sectional surveys. We examined data of eight published researches and nine cross-sectional surveys conducted during the period 2020 to end of 2021.

**Results:**

In the review of eight published studies (seven from 2020 and one from 2021) of cross-sectional type, online, with sample sizes from 155 to 904 (four with student population, two with healthcare workers and two with general population) the prevalence of anxiety was from 13% to 73% (average 47.07%; measured with HADS, DASS-21 and GAD-7) while the prevalence of depression was from 13.9% to 71.60% (average 34.42%; measured with HADS, DASS-21 and PHQ-9). The methodological quality of included studies was low. Analysis of raw data of our nine cross-sectional surveys conducted during the period 2020 to end of 2021 show pooled prevalence for anxiety (measured with GAD-7, ≥10) for 2706 participants (five samples) to be 34.59 % (95% CI, 33.11%-36.07%) and for depression (measured with PHQ-9, ≥10) for 3703 participants (seven samples) to be 40.84% (95% CI, 39.43%-42.25%). Mostly, younger age, females and low SES emerged as factors associated with higher levels of anxiety/depression.

**Conclusions:**

Our anxiety/depression prevalence rates, mostly is above average in comparison to other countries in Europe. Despite awareness for methodological flaws of studies, measurement invariances and some evidence of variation in the estimated prevalence rates of depression and anxiety disorder across countries; high prevalence of mental health problems during the pandemic indicates need for mental health prevention, promotion and intervention programs to mitigate the consequences of the COVID-19 pandemic on the population.

**Disclosure of Interest:**

None Declared

